# Continual proteomic divergence of HepG2 cells as a consequence of long-term spheroid culture

**DOI:** 10.1038/s41598-021-89907-9

**Published:** 2021-05-25

**Authors:** Andrea Antonio Ellero, Iman van den Bout, Maré Vlok, Allan Duncan Cromarty, Tracey Hurrell

**Affiliations:** 1 Department of Pharmacology, Faculty of Health Sciences, School of Medicine, University of Pretoria, Pretoria, South Africa; 2 Centre for Neuroendocrinology, Faculty of Health Sciences, School of Medicine, University of Pretoria, Pretoria, South Africa; 3 Department of Physiology, Faculty of Health Sciences, School of Medicine, University of Pretoria, Pretoria, South Africa; 4 Proteomics Unit, Central Analytical Facility, Stellenbosch University, Stellenbosch, South Africa; 5 Bioengineering and Integrated Genomics Group, Next Generation Health Cluster, Council for Scientific and Industrial Research, Pretoria, South Africa

**Keywords:** Cell biology, Protein analysis, Proteome informatics, Biological models, Mass spectrometry, Microscopy, Proteomic analysis

## Abstract

Three-dimensional models are considered a powerful tool for improving the concordance between in vitro and in vivo phenotypes. However, the duration of spheroid culture may influence the degree of correlation between these counterparts. When using immortalised cell lines as model systems, the assumption for consistency and reproducibility is often made without adequate characterization or validation. It is therefore essential to define the biology of each spheroid model by investigating proteomic dynamics, which may be altered relative to culture duration. As an example, we assessed the influence of culture duration on the relative proteome abundance of HepG2 cells cultured as spheroids, which are routinely used to model aspects of the liver. Quantitative proteomic profiling of whole cell lysates labelled with tandem-mass tags was conducted using liquid chromatography-tandem mass spectrometry (LC–MS/MS). In excess of 4800 proteins were confidently identified, which were shared across three consecutive time points over 28 days. The HepG2 spheroid proteome was divergent from the monolayer proteome after 14 days in culture and continued to change over the successive culture time points. Proteins representing the recognised core hepatic proteome, cell junction, extracellular matrix, and cell adhesion proteins were found to be continually modulated.

## Introduction

The pharmaceutical industry implements increasingly stringent standards in drug development, and enforces persistent regulatory reviews of both new drug candidates (NDCs) and commercially available drugs^1,2^. Only a small percentage of NDCs entering development fulfil the requirements to enter into Phase I clinical trials, with less than 10% becoming licenced products^3^. Failure of both candidates in development and marketed drugs impose great costs to both industries and consumers. Though numerous factors contribute to drug candidate failure or post marketed drug withdrawal, safety and efficacy together contribute 74–76%^4^. This is largely due to the limitations and inconsistencies of preclinical screening models. Exploring this, hepatotoxicity has been cited as the most common causative factor for withdrawal of post marketed drugs^5,6^. When using immortalized or primary cell cultures as surrogates for their in vivo counterparts it is important to adequately characterize each model system before being able to make reliable biological inferences. Currently used liver models which include; primary human hepatocytes (PHH), transformed hepatocytes (HepG2, Huh7, and HepaRG), and hepatocyte-like cells (HLCs) derived from embryonic or induced pluripotent stem cells, are both genotypically and phenotypically distinct due to their various origins^7–9^. Each liver model has unique limitations, including the high cost and inter-donor variability of PHH^10^, the lack of clinically relevant biotransformation capacity and strong cancer signatures of HepG2 cells^11^, or the immature and variable hepatic phenotype associated with HLCs^12,13^. These distinctive characteristics make each model fit for only certain applications. However, an advantage conferred to all in vitro liver model systems is the capacity for adaptation to three-dimensional (3D) culture, which supports the maintenance or acquisition of a more in vivo representative phenotype^14,15^. This notion of improved phenotype has been widely adopted by researchers, using cells of various origins, in attempts to recapitulate both organs and disease conditions such as tumours in vitro^16–18^. Duration of culture, presence or absence of extracellular matrix (ECM) proteins, static or perfused systems, ratio of cell number to culture media volume, and spheroid physical dimensions are all determinants of in vitro phenotypes^19^, although a complete understanding of how each influence cell behaviour and phenotype remains unclear. Shifting the paradigm from monolayer to 3D culture systems has, in most models, not sufficiently addressed the progressive changes that occur as a consequence of duration of time spent in culture. Despite the widely accepted notion that cells cultured in 3D allow for more representable phenotypes, the rationale for experimental time points is less thoroughly characterized. The impact of a dynamic baseline would be evident in experiments such as drug testing for efficacy or toxicity, if cells which rapidly form spheroids, were cultured for 7, 10, or 28 days. While recognised as limited in their biotransformation capacity^20^, HepG2 cell spheroids are used to investigate genotoxicity^21^, and predict hepatotoxicity^22,23^. Phenotypic transitions as a result of growth in 3D have been investigated at the gene transcription level^24^ which while informative, does not account for the fact that only 20–50% of transcribed genes result in expression of functional proteins^25^, which would limit the perceived utility of these model systems. HepG2 monolayers boast a highly reproducible detectible proteome, while the proteome of HepG2 spheroids cultured for 10 days inconsistently diverges from monolayers across this timeframe^26^. Therefore, since proteomic changes which occur as cells arrange and mature within complex 3D structure are often overlooked^27^, we sought to monitor the relative quantitative changes within spheroids over a long-term culture of 28 days using isobaric tagging. This was done to determine whether cells maintained in spheroids for more than 10 days undergo reproducible, significant, proteomic changes related to their altered culture architecture. These data suggest that there is a continual divergence of the HepG2 proteome as a consequence of long-term spheroid culture which impacts cellular functionality. We provide new insights into the dynamic nature of 3D culture systems and highlight important considerations essential to the field of biological modelling.

## Results

### Spheroids undergo dynamic proteomic transitions based on time spent in culture

**Figure 1:**
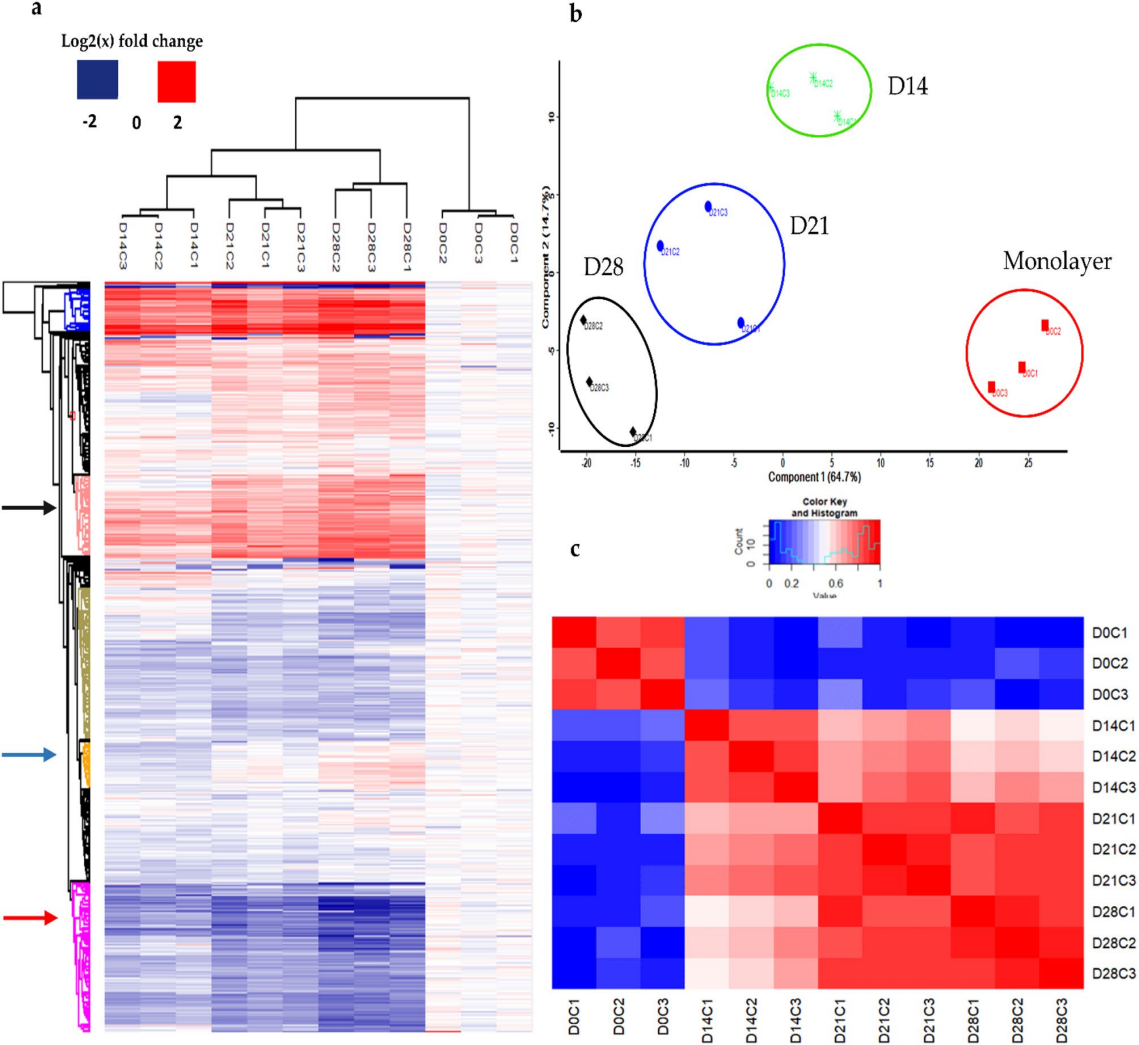
(**a**) Hierarchical clustering of proteomic data cohorts from biological replicates of monolayer (D0C1, 2, 3) and spheroid groups at Days 14 (D14C1, 2, 3), 21 (D21C1, 2, 3), and 28 (D28C1, 2, 3) in culture. (**b**) PCA of HepG2 cell monolayers (red) and spheroids at Days 14 (green), 21 (blue), and 28 (black) comparing Component 1 versus Component 2 and (**c**) correlation plot showing strong correlations between biological replicates as well as moderate correlations across successive time points, whereas poor correlations were observed between D0C monolayers and spheroid cultures from D14C, D21C, and D28C. Figures generated using Perseus v. 1.6.7.0 software^28^ and InfernoRDN v. 1.1.7626.35996 https://omics.pnl.gov/software/InfernoRDN^29^.

Successful isobaric tagging allowed for confident identification and relative quantification of 4819 proteins shared across biological replicates within experimental groups (Supplementary Table S1). Hierarchal clustering (Fig. 1a) segregated the monolayer proteome (D0C) from all three spheroid groups D14C, D21C, and D28C. Changes in the relative abundances of protein clusters (blue, black, and red arrows) correlate with culture time. These trends demonstrate continual proteomic changes within the analysed spheroid cultures, which is reproducible across biological replicates, but dynamic within the observable temporal frame.

Distinct spatial clustering in PCA (Fig. 1b), where PC1 contributes 64.7% of the variance, appears to distinguish culture methodology whereby continual separation between spheroid cultures over time is also observed. PC3 (Supplementary Fig S1), contributed only 4.4% of the variance, and was not able to spatially resolve groupings despite maintaining resolution of monolayers and spheroids by culture time along PC1. Contrary to observations in a previous study^26^, the spheroid replicates produced reproducible proteome data when cultured for longer than 14 days. Correlation plots (Fig. 1c) were poor when comparing monolayers to spheroids from each of Day 14, 21, and 28 replicates, illustrating the magnitude of proteomic divergence from monolayers. In contrast, high correlations between biological replicates of both monolayers and spheroids within the same experimental groups were observed. This high degree of correlation was seen to diminish progressively with time, when comparing spheroid replicates from day 14 to those from days 21 and 28.

**Figure 2:**
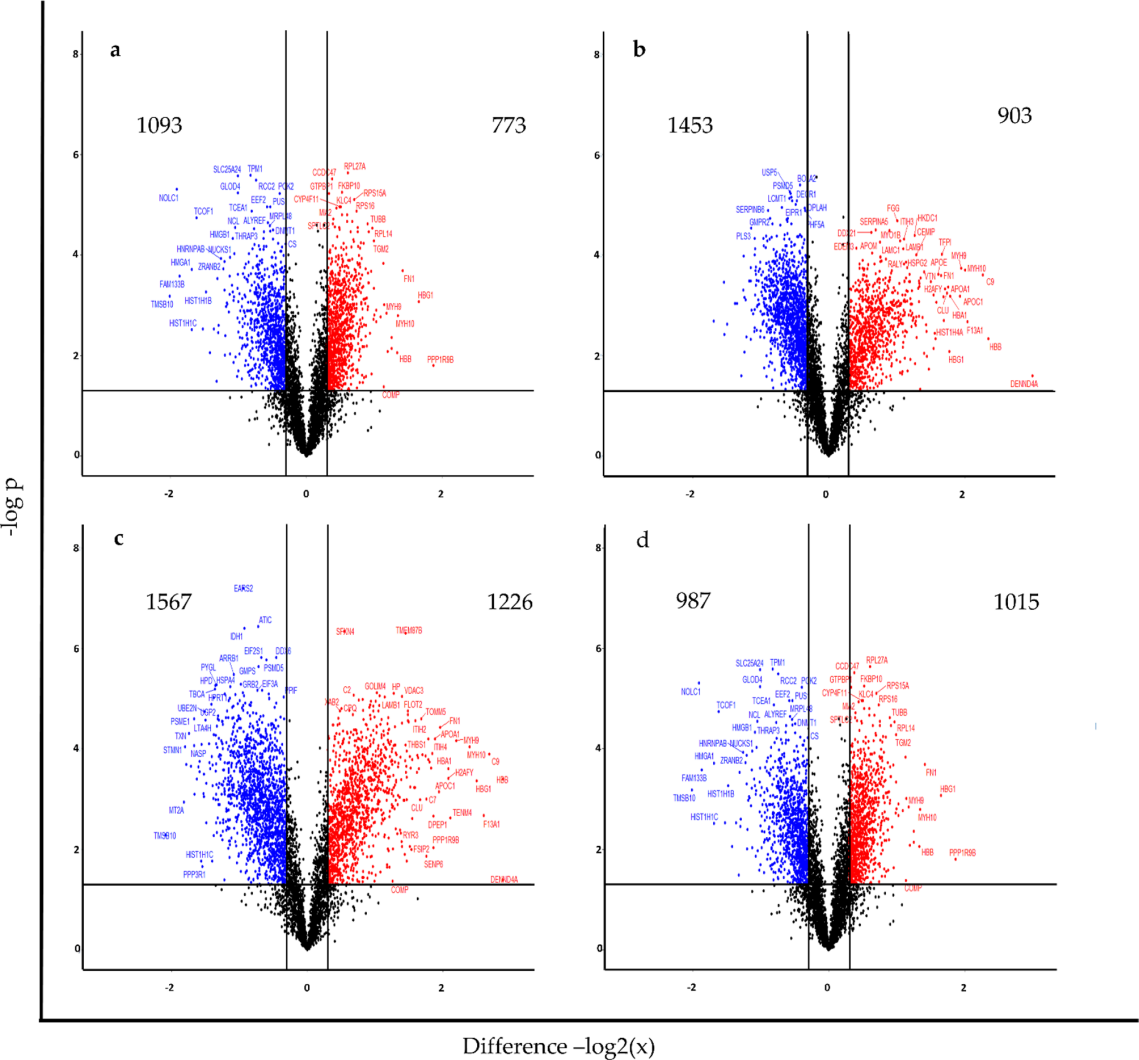
Volcano plots indicating significant differences in proteins between experimental groups identified using a two-sided t-test allowing for 250 randomisations, an FDR of 0.05 and mean fold change of log2(x) ± 0.3 for (**a**) D14C verses D0C, (**b**) D21C verses D0C, (**c**) D28C verses D0C, and (**d**) D28C verses D14C. Figure generated using RStudio software loaded with the ggplot package^30,31^.

To determine the number of differentially abundant proteins, volcano plots were generated using a two-sided t-test with an FDR of 0.05 while allowing for 250 randomisations and mean − log2(x) fold change of ± 0.3. Proteome data of spheroids from sequential weekly time points were compared to baseline monolayer cultures (D0) (Fig. 2a, b, c; Supplementary Tables S2-S4). Additionally, Day 14 (D14C) spheroid proteomes were compared with those of Day 28 (D28C) spheroids (Fig. 2d; Supplementary Table S5). Using these significance thresholds, the proportion of differentially abundant proteins increased with spheroid culture duration. At Day 14, 38.72% of the detected proteome had superseded statistical thresholds compared to monolayers, and this number increased along the temporal frame to 48.89% at Day 21 and 57.96% at Day 28. Furthermore, the changes between Day 14 and Day 28 involved 41.54% of the observed proteome indicating that the spheroid proteome continues to evolve over time.

### Spheroids remain viable over a 28-day time course with a progressive increase in abundance of hepatic proteins relative to duration in culture

**Figure 3:**
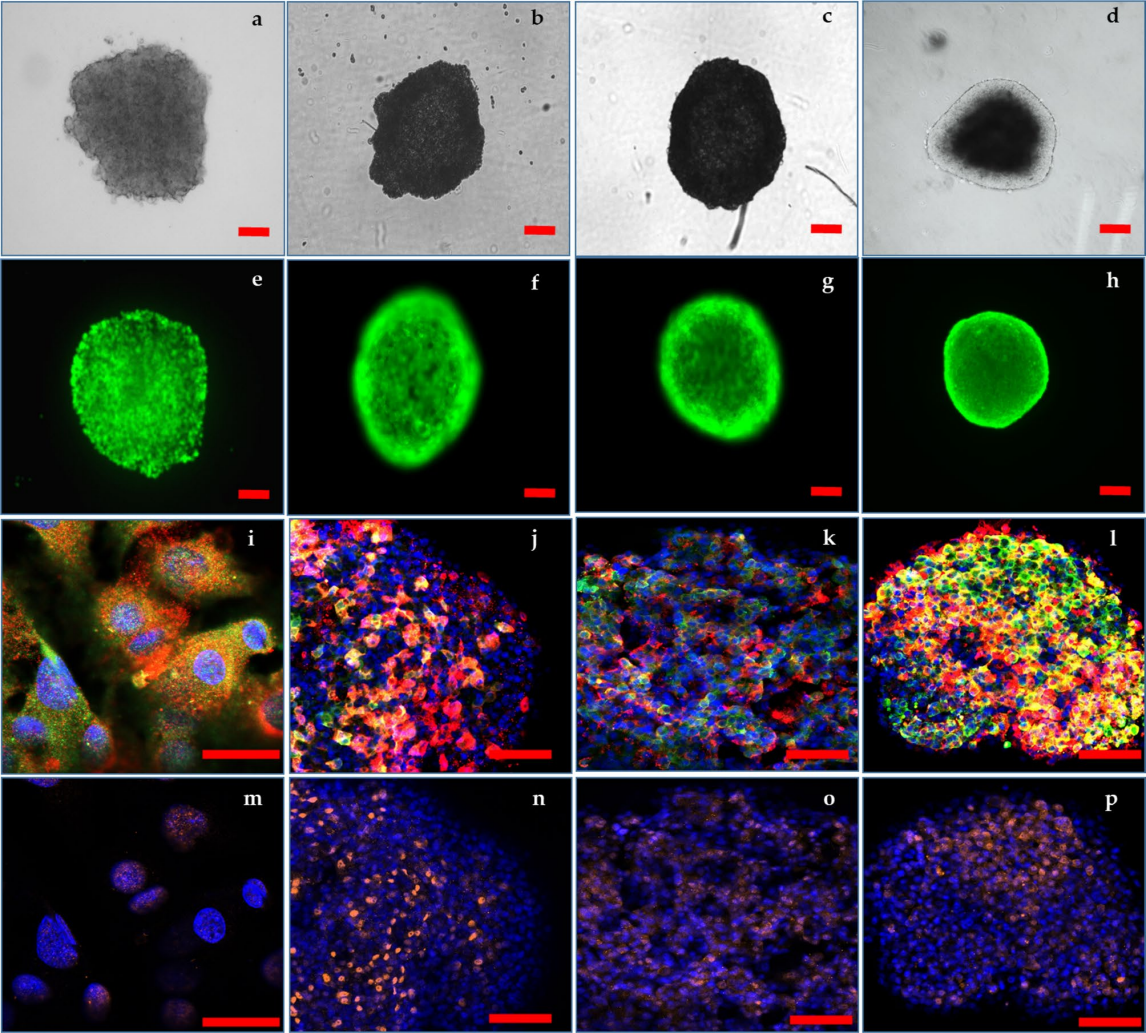
Images of the progression of HepG2 spheroids over a time course of 6 weeks in culture. Light micrographs depicting HepG2 spheroids at Days 14 (**a**), 21 (**b**), 28 (**c**), and 42 (**d**) post-seeding. Fluorescent micrographs of HepG2 spheroids at Days 14 (**e**), 21 (**f**), 28 (**g**) and 42 (**h**) stained with FDA and PI showing maintenance of esterase activity and no evidence of compromised cell membranes. Confocal images of cleared HepG2 monolayers (**i**) and cleared spheroids at Days 14 (**j**), 21 (**k**), 28 (**l**), stained with DAPI (blue), ALB (green) and AFP (red). Confocal images of HepG2 monolayers (**m**) and cleared spheroids at Days 14 (**n**), 21 (**o**), 28 (**p**) stained with DAPI (blue) and HNF4α (orange). Scale bars 100 µm for spheroids and 20 µm for monolayers. Generated using Zeiss Zen Blue edition software 3.0 (https://www.zeiss.com/microscopy/int/products/microscope-software/zen.html).

Protein content per spheroid (Supplementary Fig S2) was quantified throughout the time course as an inference for cellular biomass. Increases in protein content were greatest within the first two weeks of culture but then plateaued from Days 14 to 28. Spheroids were monitored for morphological features including structural integrity and compactness (Fig. 3a–d) as well as maintenance of esterase activity (Fig. 3e–h, Supplementary Fig S3, Supplementary videos S1-S2 (positive control for cell death)) as an indicator of cell viability. While the core proteomic assessments in this study were conducted over 28 days, these features were measured over a period of up to 6 weeks in order to demonstrate the long-term viability potential of this model system. HepG2 spheroids expressed hepatic marker proteins albumin (ALB), α-fetoprotein (AFP), and hepatic nuclear factor 4 alpha (HNF4α), as evidenced by confocal imaging (Fig. 3i–p; Supplementary videos S3-S5). Quantitation of AFP and ALB staining (Supplementary Fig S4) supported the trends in the TMT-labelled datasets (Supplementary Table S1). HNF4α is a master regulator for hepatocyte differentiation^32^ and is essential for the expression of other hepatic transcription factors and for maintaining hepatocyte function^33,34^, which remained consistently expressed throughout the time course. AFP, a glycoprotein derived from embryonic endoderm cells of foetal liver, decreases in abundance throughout hepatic maturation^35^ whereafter ALB progressively increases in abundance in mature livers^36^. AFP and ALB increased in abundance in HepG2 spheroids, which given the cellular origin of these cells, as being from a well-differentiated hepatocellular carcinoma, the progressive increase in AFP expression is expected^37^.

**Figure 4:**
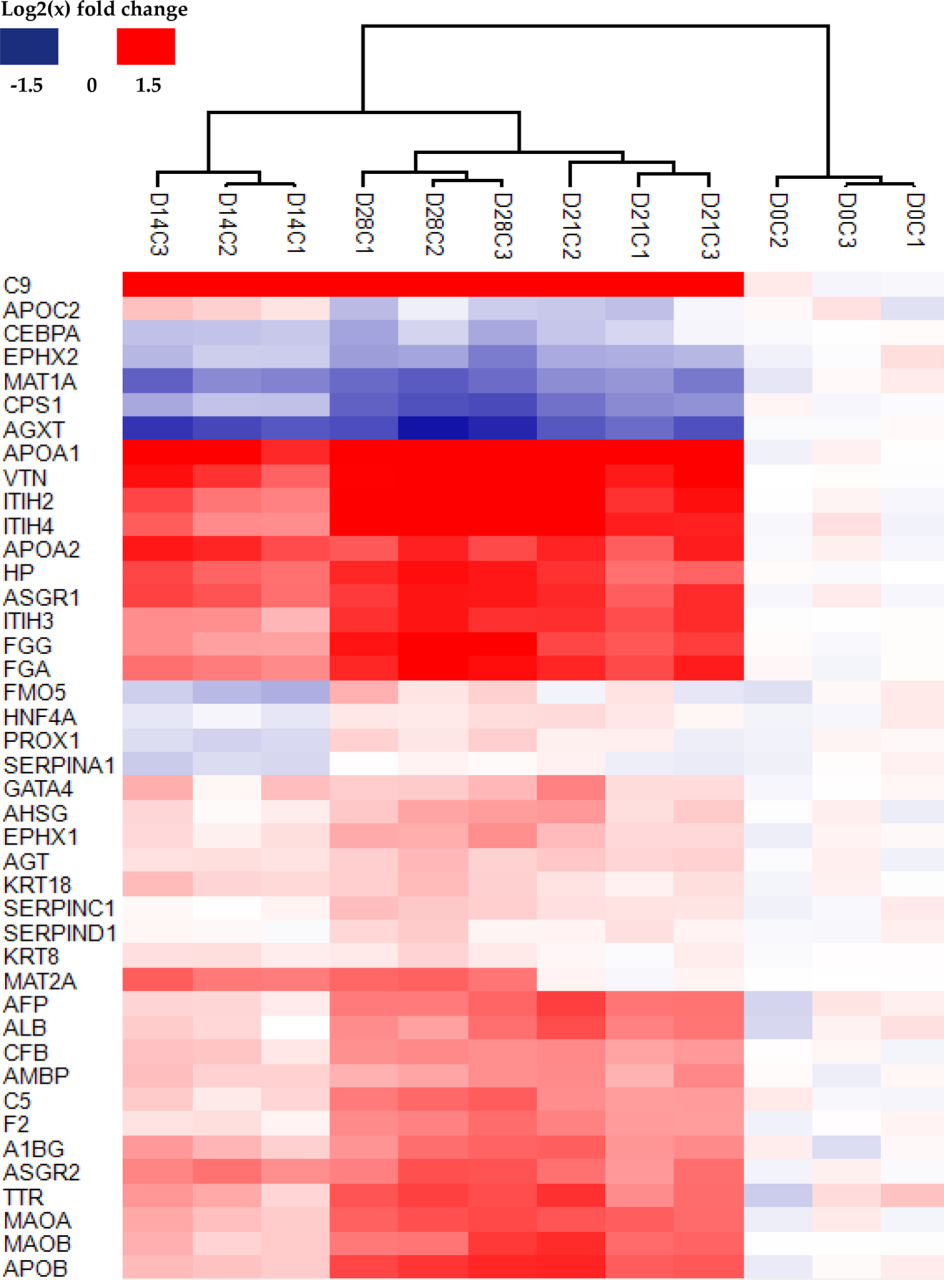
Hierarchical clustering of enriched proteins representative of a hepatic phenotype. Figure generated using Perseus v. 1.6.7.0 software^28^.

The relative quantitative proteomic data generated allows for not only a global overview of the divergence of monolayer and spheroid cultures but also for interrogating the abundance of enriched protein data sets which underlie these differences. Spheroid culture improves the expression of cell specific markers, suggesting that cells within spheroids become functionally closer to their in vivo counterparts^38–40^, which has been observed in numerous hepatic cell spheroid cultures^12,13,24^. The potential for bias in the protein expression patterns within the enriched data sets due to the cellular origin of these spheroids could be a limitation. Therefore, datasets from this study were searched against a list of proteins which correlates with hepatic phenotypes from previous studies^11,13,41^, and then enriched for analysis. Expression of proteins typically followed a progressive pattern whereby if a protein is up or down regulated at Day 14 this change continued through to Day 28 (Fig. 4, Table 1; and Supplementary Table S6). Several proteins exhibited reduced abundance in spheroids when compared to the monolayer cells, namely; CEBPA (enhancer-binding protein alpha), EPHX2 (bifunctional epoxide hydrolase 2), MAT1A (adenosylmethionine synthase isoform type-1), CPS1 (carbamoyl-phosphate synthase), and AGXT (pyruvate aminotransferase). These proteins have a variable expression profile within hepatocellular carcinoma and liver tissue. Decreased AGXT expression is implicated in the progression of hepatocellular carcinomas^42^, while CEBPA has a role in the regeneration of normal livers and typically shows down-regulated levels of its mRNA expression during growth of freshly isolated hepatocytes^43^. This data shows that HepG2 cells express some hepatocyte marker proteins more abundantly when cultured as spheroids for more than for 21 days, suggesting that they could attain a more hepatocyte-like proteome than that achieved during monolayer or early spheroid culture. Indeed, upregulated proteins fulfil a variety of hepato-specific functions such as those acting as nuclear factors (HNF4α and GATA4), apolipoproteins (APOA/B/C variants), and cytokeratins expressed in bile canaliculi of mature hepatocytes (KRT18). Their relatively increased abundance may still be insufficient, or conversely exceed what is observed within whole liver tissue but provides insight into the continual proteomic changes taking place during the time-course of spheroid culture. Cohorts were further enriched for proteins involved in hepatic drug metabolism (Supplementary Table S7) using the “core” and “extended” Absorption, Distribution, Metabolism and Excretion (ADME) lists (http://pharmaadme.org/; Supplementary Table S8) This ADME protein cohort was poorly represented in the dataset, with only 94 proteins of the combined list of 299 proteins present. This could be due to the poor metabolic competence of these cells, or alternatively due to a relatively low abundance of these proteins within whole cell lysates. Where present, these proteins exhibited a variable expression change with time (Supplementary Fig S5).

**Table 1:** Expression of proteins representative of a hepatic phenotype as compared to monolayers.

Gene names	T-test significant D14C	T-test significant D21C	T-test significant D28	Mean D14C	Mean D21C	Mean D28C
C9	+	+	+	1.91	2.27	2.69
APOC2				0.25	− 0.24	− 0.25
CEBPA	+		+	− 0.33	− 0.2	− 0.41
EPHX2	+	+	+	− 0.32	− 0.44	− 0.59
MAT1A	+	+	+	− 0.74	− 0.65	− 0.85
CPS1	+	+	+	− 0.39	− 0.68	− 0.94
AGXT	+	+	+	− 1.02	− 0.91	− 1.16
APOA1	+	+	+	1.41	1.75	1.88
VTN	+	+	+	1.11	1.50	1.63
ITIH2	+	+	+	0.82	1.33	1.59
ITIH4	+	+	+	0.72	1.40	1.86
APOA2	+	+	+	1.16	1.12	1.05
HP	+	+	+	0.89	0.93	1.27
ASGR1	+	+	+	0.93	1.08	1.22
ITIH3	+	+	+	0.55	1.10	1.19
FGG	+	+	+	0.56	1.01	1.44
FGA	+	+	+	0.72	1.15	1.39
FMO5	+			− 0.37	− 0.02	0.28
HNF4A			+	− 0.11	0.13	0.15
PROX1	+		+	− 0.21	0.03	0.22
SERPINA1	+			− 0.24	− 0.04	0.03
GATA4			+	0.29	0.36	0.32
AHSG			+	0.12	0.35	0.45
EPHX1			+	0.16	0.26	0.51
AGT	+	+	+	0.17	0.26	0.30
KRT18	+		+	0.28	0.14	0.31
SERPINC1		+	+	0.03	0.16	0.31
SERPIND1				0.02	0.10	0.19
KRT8	+			0.15	0.04	0.16
MAT2A	+		+	0.79	0.03	0.82
AFP		+	+	0.19	0.87	0.77
ALB		+	+	0.17	0.81	0.65
CFB		+	+	0.27	0.58	0.63
AMBP	+	+	+	0.29	0.58	0.52
C5		+	+	0.21	0.57	0.82
F2		+	+	0.13	0.59	0.72
A1BG		+	+	0.41	0.70	0.75
ASGR2	+	+	+	0.69	0.72	0.88
TTR		+	+	0.43	0.86	0.99
MAOA	+	+	+	0.37	0.88	0.95
MAOB	+	+	+	0.32	0.95	0.86
APOB	+	+	+	0.33	1.01	1.11

^1^Values reported are the means, as Log2(x), of three biological replicate experiments showing relative fold change relative to monolayer counterparts. + indicates significance for q-value ≤ 0.05. Original non-averaged values may be found in Supplementary Table S1.

### Curating biological processes to assess spheroid dynamics

In large proteomic datasets, biologically significant changes are not always of sufficient magnitude to reach statistical thresholds set prior to analysis. Spheroid cultures increase the proportion of cells in direct contact with each other. Intuitively, when considering the possible drivers of proteomic changes, the most likely contributors are the proteins which facilitate these cell–cell interactions i.e. extracellular matrix (ECM) (GO:0031012), proteins which facilitate cell–cell attachment and regulate intercellular signalling pathways^44^, which are also associated with cell junction proteins (GO:0005911), and cell–cell adhesion molecules (GO:0098609). Therefore, the trends for proteins of these classes were investigated. To avoid missing potentially important divergent proteins or biasing based on statistical thresholds, protein annotation and functional classification was used, where proteins were annotated and filtered based on the associated GO terms, and enriched using the PANTHER gene function classification system^45^.

**Figure 5:**
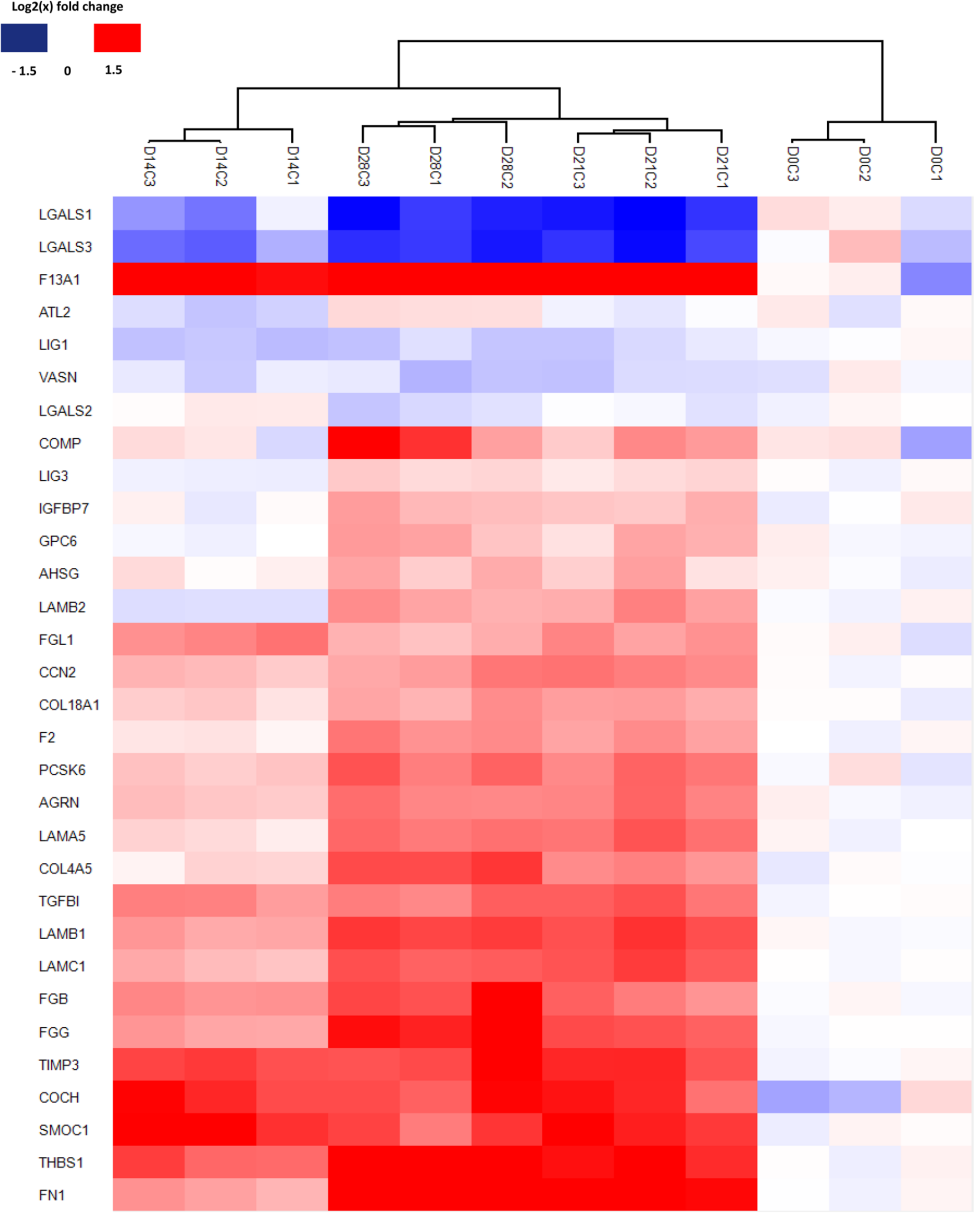
Hierarchical clustering of proteins involved in the ECM. Figure generated using Perseus v. 1.6.7.0 software^28^.

Major ECM proteins (Fig. 5; Supplementary Table S9) increased in abundance in spheroid cultures, seemingly increasing with time. The greatest fold changes were observed for various functional proteins produced by the liver; coagulation factor XIII (F13A1), tissue inhibitor of metalloproteinase 3 (TIMP3), and thrombospondin (THBS1) with notable changes also evident in laminin (LAMB1/2 and LAMC1) proteins. Galectin proteins 1 and 3 (LGALS1 and LGALS3) significantly decreased in expression over the time course contrary to what has been reported for hepatocellular carcinomas^46^. Interestingly, when enriching for proteins involved in focal adhesions (Supplementary Fig S6; Supplementary Table S10), integrin beta 1 (ITGB1) was found to be one among only 4 proteins of this class with a significant increase in abundance through the time course of spheroid culture. Previous evidence has shown that the expression of ITGB1 is required for the expression of LGALS1^47^, and despite the increased ITGB1 expression, LGALS1 was progressively decreased in spheroids. TIMP3 has been shown to have its expression silenced in hepatocellular carcinoma^48^, contrary to the trend being observed in this study. Literature has reported that increased expression of specific laminin proteins is integral to the differentiation of stem cells into hepatocyte-like cells whereby progression of feto-hepatic phenotypes to a more mature hepatic phenotype is promoted^49^.

Cell–cell junction (Supplementary Fig S7; Supplementary Table S11) and cell–cell adhesion proteins (Supplementary Fig S8; Supplementary Table S12) are involved in cell-substrate and intercellular attachment and regulate critical pathways such as monitoring of barrier functions in epithelia and playing critical roles in cell proliferation and cellular migration^50^. Integrins are the main cell-extracellular matrix adhesion molecules involved in the formation of focal adhesions and hemidesmosome junctions, while cadherins are usually involved in cell–cell adhesion molecules that forms adherens and desmosomal junctions. These anchoring junctions regulate the roles of intercellular molecules such as actin and intermediate filaments by dictating the cytoskeletal structure of the cell. Cx32, a protein that forms gap junction channels for cell–cell communication^51^, is progressively up-regulated throughout the time course. Though the abundance of proteins involved in cell–cell junctions and adhesions varied, where increased or decreased abundance was observed for a specific protein, this progression was again relative to duration in culture.

## Discussion

Cell culture is integral in the development of therapeutics and continues to advance the understanding of biological pathways. The continual evolution of in vitro modelling has in recent times seen 3D cultures being widely adopted for new applications with several underlying assumptions being accepted. Spheroids allow for better biological mimicry of in vivo tissues when compared to monolayers^52^, especially in the case of hepatocyte derived models^53,54^. Furthermore, unlike monolayers which are generally limited in culture duration from seeding to confluence, spheroids may be cultured in excess of 5 weeks if given optimal conditions. This greatly extended culture time necessitates that the focus shift to whether a certain model is the most appropriate platform to answer a certain research question but also requires assessing at which point the model can most accurately and reproducibly do so? This study presents a model for HepG2 cell culture which has, within set statistical parameters, highlighted a complete divergence of the cellular proteome of spheroid cultures from that of monolayers, contrary to what has been previously observed when assessing spheroids cultured for a duration of 10 days^26^. This divergence takes place reproducibly across biological replicates, within this system, if the spheroids were cultured for a minimum of 14 days from seeding. The multidimensional cell-to-cell interactions and improved paracrine signalling resulting from spheroid culture allows for new gene expression pathways to be activated and ultimately allows for a divergence in cellular phenotype in cells receiving these signals^55,56^. The magnitude of this divergence is not well investigated*,* nor is it frequently reported in literature. Cells within spheroids progressively deposit extracellular matrix, thereby altering the abundance of proteins involved in cell junctions and cell adhesion^57^. The ECM is an intricate network comprising of multiple proteins, many of which provide important information for the building of a sophisticated structure required for anchoring cells and sustaining normal function of tissues. The matrix itself has been reported to be considered as a paracrine/endocrine entity, with more complex functions than previously appreciated^58^. Additionally, in the case of hepatocytes, the mechanisms by which these cells attach, changes from being largely integrin mediated in monolayer culture to being facilitated by cadherins and extracellular matrix proteins, which drives further phenotypic changes^59^. A separate consideration, not addressed here, is the variability with which cells from different origins form spheroids and how universal these time-dependent proteomic changes are across different cell type models.

As reported before^26^, spheroid proteomes do indeed diverge from monolayers, however, the time taken for this divergence to occur reproducibly across replicates was not consistent over the 10-day time course. Therefore, the culture period required to reach a stable or more biologically applicable phenotype is critical to the appropriate timing of experimentation using these models. Following this narrative, prior to acceptance as biological models, within academia or industry, cell phenotypes need to be thoroughly characterised. In this study it was observed that while the proteome of spheroid cultures converges with time, the equilibrium reached is still not static but rather dynamic, and dependant on culture time. This makes standardisation challenging and researchers need to extensively characterise culture models with this consideration in mind. The focus for the development of 3D culture methodologies should be to develop reproducible and predictable ‘fit-to-purpose’ assays or models. While no single model currently exists to perfectly recapitulate the in vivo scenario in its appropriate complexity, the use and development of appropriate and validated models remains paramount to basic biological sciences.

Although gene expression changes are typically several orders of magnitude higher than proteomic variances^60^, the consensus and quality of the abundant protein cohort in spheroid cultures demonstrates that large scale proteomic changes should be used to characterize and define spheroid dynamics and not only evidenced at the transcript level. However, defining these ‘fit-to-purpose’ models requires the consideration that large fold changes in protein levels are not always essential to bring about biological changes, and that what is significant in a statistical sense is not always significant in a biological sense, which is difficult to discern within global proteomic datasets.

As presented here, cells of a single origin are capable of undergoing significant changes to the proteome even when keeping all external conditions consistent and introducing a simple modulation of the culture environment over time. The changes observed do not fully endorse the narrative that spheroid culture allow for a more representative model of the in vivo scenario. While these cells are derived from a hepatocellular carcinoma the changes observed do not fully fit with the notion that the phenotypes of these spheroids become more like a hepatocellular carcinoma. While some cancer signature proteins are indeed increased as in the case of AFP (Fig. 3g–i, 4; Table 2). Others such as TIMP3 (Fig. 4), AGXT and LGALS1/3 (Fig. 5) changed in the favour of a non-cancerous hepatic phenotype. These changes were coupled to a general increase in the abundance of hepatic marker proteins. Proteome changes of this nature, whether considering this platform to model either hepatotoxicity testing or hepatocellular carcinoma cytotoxicity, cannot be overlooked.

**Table 2:** Primary and secondary antibodies for immunofluorescent staining.

	Species	Vendor	Dilution
**Primary antibody**			
Albumin	Chicken	Abcam	1/100
HNF4α	Rabbit	Abcam	1/100
α-fetoprotein	Mouse	Abcam	1/50
**Secondary antibody**			
Alexa Fluor 488	Goat anti-chicken	Abcam	1/1000
Alexa Fluor 555	Donkey anti-rabbit	Abcam	1/1000
Alexa Fluor 647	Donkey anti-mouse	Abcam	1/1000

The time course presented here is limited to 28 days, and as noted from the observations presented in Fig. 1, these changes remain dynamic making it unclear as to how long this progression could continue as well as how beneficial or detrimental it would be to cellular functionality. Additionally, dissociation by TrypLE may have biased the baseline for cellular membrane bound proteins in monolayers as compared to spheroids. However, as the same monolayer served as the Day 0 protein sample and was used to seed the spheroids, the changes in the proteome over days 14 to 28 successfully characterize the temporal dynamics of the spheroid proteome. Despite these uncertainties, spheroid models such as this are central to research in the pharmaceutical industry and academia^14,61^, and the critical task remains to interrogate long term spheroid dynamics and validate these models as appropriate for the assays for which they are intended.

This study provides insights into the dynamic nature of 3D culture systems and highlights important considerations essential to the application of a biological model. We have previously reported that spheroids cultured for 10 days in hanging-drops have a less reproducible proteome, across biological replicates, when compared with monolayers. Here it was demonstrated that increased culture time allowed for a continual divergence of the proteome of spheroid cultured cell replicates from monolayer cultures. There is a continuous, reproducible, divergence of proteome along the temporal frame with regards to proteins associated with hepatic phenotype, cell junctions, extracellular matrix, and cell-adhesion molecules. Taken together, these data demonstrate how highly dynamic the proteome of spheroid cultures are and provides a resource for assessing proteomic changes based on cell culture modulation. Adequate understanding of time-dependent changes derived from modulation in cell culture methodology in spheroid cultures is essential in reducing proteomic heterogeneity and may ultimately allow for these ‘fit-to-purpose’ models to become reproducible even across laboratories.

## Materials and methods

### HepG2 cell culture and spheroid formation

Human hepatoma HepG2 cells were obtained from Cellonex (Johannesburg, RSA: CHG2-C) and cultured in Dulbecco’s modified minimum essential medium (DMEM) supplemented with 10% foetal bovine serum (FBS), and 2 mM GlutaMAX. HepG2 cells were thawed and cultured to 80% confluence (5–6 days) for 2 passages prior to harvesting monolayer controls (D0) using TrypLE and seeding spheroids. Spheroids were generated by seeding 20,000 cells/well in 45 µl of medium into Perfecta3D 96-well hanging drop plates (3D Biomatrix; Michigan, USA) according to the manufacturer’s protocol. Cells aggregated under gravity at the droplet apex to form a single spheroid per well and partial exchange of growth medium (12 µl) was conducted every alternate day. Spheroids were harvested for analysis at culture Days 14, 21, and 28.

### Sample collection and protein quantitation

Cells from dissociated monolayers were harvested as the Day 0 control. Cells were washed with PBS and lysed using 100 µl lysis buffer (10 mM Tris-HCL (pH 8), 1 mM ethylene diaminetetraacetic acid, 0.5 mM ethylene glycol tetraacetic acid, 1% Triton X-100, 0.1% sodium dodecyl sulphate, 0.1% sodium deoxycholate, 140 mM sodium chloride) containing complete protease inhibitor cocktail. Cell lysis was aided by ultrasonic disruption on ice (1500 W, 5 min), and an additional 15 min incubation on ice, after which the lysate was centrifuged at 16,000*g* for 10 min at 4 °C. Soluble protein was quantified using the bicinchoninic acid assay. Absorbance was determined using a Bio-Rad iMark microplate reader at 560 nm. Spheroids (n = 80) were collected, combined, and homogenised in 300 µl lysis buffer using a Dounce homogeniser prior to protein quantitation as described above.

### Protein preparation and isobaric tag labelling

Biological replicates (n = 3) of HepG2 monolayers (D0), HepG2 spheroids at Day 14 (D14), Day 21 (D21), Day 28 (D28) and a pooled group comprised of equivalent amounts of protein from each experimental group (Pool) were each labelled with one of the 6-plex tandem mass tags (TMT; Thermo Scientific; Maryland, USA). Seventy-five micrograms (75 µg) of protein was reduced at 37 °C for 1 h using 10 mM dithiothreitol, then alkylated at room temperature with 25 mM iodoacetamide. Proteins were precipitated overnight at 4 °C after addition of 10 volumes of 100% acetone, harvested by centrifugation at 16,000*g* (20 min) and resuspended in 100 mM HEPES (pH 8.5). Samples were digested with sequence-grade modified trypsin (1:40) for 1 h at 37 °C, with a further overnight digestion at 37 °C after adding additional trypsin (1:40). Each TMT tag was resuspended in 41 µl mass spectrometry-grade acetonitrile. Supernatants from clarified (20 min; 16,000*g*) digested peptides, were labelled for 2 h at room temperature under constant agitation. Labelling was quenched by adding 8 µl of 5% hydroxylamine for 1 h and incubated overnight at 4 °C with dH_2_O^62^. To circumvent tag affinity bias, experimental group’s biological replicates were divided across tag sets and randomized across the 6-plex tag sets. The different tag labelled samples were combined to contain all corresponding 6-plex tags and dried under vacuum centrifugation.

### Solid phase extraction and peptide fractionation

The combined labelled peptides were solubilized in dH_2_O with 0.1% trifluoroacetic acid and loaded onto a conditioned SepPak C18 cartridge (100 mg), desalted and eluted in 70% acetonitrile with 0.05% acetic acid. Eluted peptides were vacuum centrifuge dried and resuspended in 100 µl of 20 mM ammonium formate (pH 10) with 4% acetonitrile. Sample complexity was reduced by peptide fractionation using a Shimadzu HPLC system coupled to a photodiode array detector. Priming was done using a 1:1 ratio of mobile phase A (20 mM ammonium formate buffer, pH 10) to mobile phase B (80% acetonitrile, 20 mM ammonium formate) for 10 min at 1 ml/min before reducing to 5% mobile phase B. Peptides were loaded via a single partial loop injection, onto an Ascentis C18 HPLC column (octadecyl bonded phase; 150 mm × 4.6 mm, 5 µm; pore size 100 Å). Peptides were eluted at a flow rate of 1 ml/min using an initial isocratic low organic mobile phase (5% B) after which the organic phase was increased using a multi-step gradient up to 60% B over a total run of 75 min with 1 min fractions collected. Collected fractions were pooled to approximately similar peptide abundance by combining peptides of different retention times into 13 fractions and dried by vacuum centrifugation.

### Mass spectrometry

Labelled samples were analysed (Central Analytical Facility, University of Stellenbosch) using a Dionex Ultimate 3000 RSLC nano LC (Thermo Scientific; Massachusetts, USA) system coupled to a Thermo Scientific Fusion Orbitrap Mass Spectrometer equipped with a Nanospray Flex ionization source. Peptides (1–2 µg) were loaded (mobile phase A: 2% acetonitrile with 0.1% formic acid) onto a C18 trapping-column (Thermo Scientific; 5 mm × 300 µm, 5 µm; pore size 100 Å) and a Luna C18 analytical column (Phenomenex; 350 mm × 75 µm, 3.6 µm). Samples were loaded onto the trap column at a loading-pump flow rate of 15 µl/min from a temperature controlled autosampler (7 °C) for 5 min before eluting onto the analytical column. Peptide separation was performed at 50 °C, at a flowrate of 500 nl/min using a non-linear gradient of 2–50% mobile phase B (100% acetonitrile) over 105 min. MS2 acquisition was performed using monoisotopic precursor selection for ions with charge states between 2+ and 6+. Undetermined charge states and charge states > 24 were excluded. Dynamic exclusion was conducted with mass error tolerance of ± 10 ppm with isotopes excluded after 1 time. Intensity threshold was set at 5.0e4. Scans were collected in data dependent mode with a 3 s cycle time. Selected precursor ions were fragmented by higher-energy collisional dissociation set at a normalized collision energy (NCE) of 38% in the quadrupole mass analyser and then excluded from fragmentation once for 30 s. Fragment ions (MS2) were detected in the orbitrap mass analyser (resolution: 60 000), using centroid mode, with the automatic gain control (AGC) target of 1.0e5 and maximum ion injection of 120 ms.

### Data processing and analysis

Raw spectrum files were loaded into proteome discoverer version 1.4.1.14 (Thermo Scientific, USA) and spectra were filtered using a minimum and maximum precursor mass of 350 and 5000 Da respectively with a threshold peak count of 15. Precursor and fragment masses were set to 20 ppm and 0.02 Da respectively with a maximum of 2 missed tryptic cleavages permitted. Dynamic modifications allowed for oxidation of methionine as well as deamination of glutamine and asparagine. Static modifications included Carbamidomethyl + 57.021 (C) Da, TMT 6-plex (K) Lysine + 229.163 Da, and TMT 6-plex (N-terminus). Peak lists were searched against a UniProtKB/Swiss-Prot human database (*Homo sapiens*, Canonical sequences, November 2018, Sequences: 20 194) concatenated with the common Repository of Adventitious Proteins (cRAP) using a sequential alternating SequestHT/MSAmanda search engine schema including added amino acid modifications for each new cycle. Files from Proteome Discoverer software (.msf) were imported into Scaffold Proteome Software^63^ for data validation using X!Tandem. Final spectrum and peptide matching validation was conducted using Peptide Prophet and Protein Prophet Algorithms with the false discovery rates (FDR’s) for protein and peptides set to 1% and 0.1% respectively. Relative quantitation was performed using the reporter ions quantifier built into Scaffold with the averaged D0 replicate samples set as the reference group. Proteins with missing values and not identified with at least 3 unique peptides were filtered out. Those passing validation were further analysed using InfernoRDN v. 1.1.7626.35996 (https://omics.pnl.gov/software/InfernoRDN^29^), Perseus v. 1.6.7.0 software (Max Planck Institute of Biochemistry)^28^ and RStudio^30^. For analysis conducted within Perseus and RStudio, data were transformed to log2(x) and normalised to the D0 experimental group. Global dataset statistics were conducted and group variations, standard deviations, protein ratios, and associated confidence scores were assigned. Hierarchical clustering was performed by clustering protein groups using Euclidean distances, and principal component analysis (PCA) was done using the Benjamini–Hochberg cut-off method with a 0.01% FDR. Volcano plots were generated using two-tailed t tests and mean fold change of log2(x) 0.3 as well as an FDR of 0.05 used to assign significance (q value). Correlation plots were generated using InfernoRDN, to allow all experimental groups to be relatively correlated, global normalisation of expression data from D0C, D14C, D21C and D28C was done using a reference group. Post normalisation, data was transformed log2(x) before protein replicates were imported as expression files and correlation plots generated. Annotations were assigned to protein accession numbers for gene ontology (GO), biological processes (BP), molecular functions (MF), cellular components (CC). Other analysis was conducted using GraphPad Prism windows software V7.0.0 (www.graphpad.com).

### Spheroid fixation and immunofluorescent based staining

HepG2 cells grown in monolayers were harvested, as described above, and seeded (60,000 cells/well) onto 12 mm coverslips coated with 500 µl Corning Matrigel Growth Factor Reduced (diluted 1/30 in pre-chilled DMEM) in 48-well plates. Cells at 70–80% confluence, were prefixed with 500 µl of 4% paraformaldehyde (PFA) for 10 min and then fixed in 4% PFA with 8% sucrose for 40 min. Coverslips were washed 3 times with PBS before cell permeabilization and blocking in B-PBT (1% Triton X-100, 10% FBS, and 4% bovine serum albumin in PBS) for 30 min. Samples were incubated with primary antibodies diluted in B-PBT (Albumin, HNF4α, and α-fetoprotein; Table 2) for 2 h followed by 3 washes in B-PBT before incubating with secondary antibodies (Table 2) for 2 h. Whole spheroids were harvested by pipette aspiration and fixed in 4% PFA with 8% sucrose overnight. Spheroids were washed 3 times for 1 h in PBS on a plate shaker at 100 rpm. Permeabilizing and blocking was conducted as above in B-PBT for 2 h. Spheroids were incubated with selected primary antibodies (Albumin, HNF4α, and α-fetoprotein; Table 2) in B-PBT overnight followed by washing twice for 2 h in 0.2% PBT (0.2% Triton X-100 in PBS) and one wash in B-PBT for 2 h before incubating in secondary antibody (Table 2) in B-PBT overnight. Separate samples were LiveDead stained with fluorescein diacetate (FDA) and propidium iodide (PI) as described previously^64^. Spheroids exposed to 10 µg/ml Puromycin for 16 h were used as a positive control for live-dead staining.

### Tissue clearing and microscopy

Tissue clearing was performed using an adapted version of the ClearT2 method^65^. Briefly, antibody labelled spheroids were incubated, with agitation, in 25% formamide and 10% polyethylene glycol 6000 (PEG) in water. After 10 min the solution was changed to 50% formamide, 20% PEG and incubated for 2 h replenishing every 30 min until spheroid opacity diminished. Spheroids were washed briefly in PBS before mounting on a microscope slide, with a 0.12 mm spacer, using ProLong Diamond Antifade Mountant with DAPI overnight. Spheroids were then imaged using a Zeiss LSM 800 confocal microscope using a 20 × objective. The aperture was set to 1 Airy unit and z-stack images were collected for each laser channel used (488 nm, 555 nm, 647 nm). Distances between z-stack image acquisitions were optimized per sample. Fluorescence and light microscopy were conducted using a Zeiss AxioVert A1 fitted with a Zeiss AxioCam digital camera. Images were analysed using Zeiss Zen Blue 3.0 software. Fluorescent signal from images was quantitated using the “Measure” tool built into the Zen Blue software package and normalised to relative fluorescence measurements to DAPI.

## Supplementary Information

Supplementary Video 1.

Supplementary Video 2.

Supplementary Video 3.

Supplementary Video 4.

Supplementary Video 5.

Supplementary Information 1.

Supplementary Information 2.

## Data Availability

The mass spectrometry proteomics data have been deposited to the ProteomeXchange Consortium via the PRIDE partner repository with the dataset identifier PXD024353 and https://doi.org/10.6019/PXD024353.
