# Supplementary material for: Continual proteomic divergence of HepG2 cells as a consequence of long-term spheroid culture
Source: Sci Rep. 2021 May 25;11:10917. doi: 10.1038/s41598-021-89907-9 (PMC8149451; doi:10.1038/s41598-021-89907-9)
Supplement: Supplementary file 6 — Supplementary Information 1. [file 41598_2021_89907_MOESM6_ESM.pdf]

Supplementary Figures

Continual proteomic divergence of HepG2 cells as a consequence of long-term spheroid culture

AA Ellero[1, 2], I van den Bout[2, 3], M Vlok[4], AD Cromarty[1], T Hurrell[5*]

[1] Department of Pharmacology, Faculty of Health Sciences, School of Medicine, University of Pretoria, South Africa
[2] Centre for Neuroendocrinology, Faculty of Health Sciences, School of Medicine, University of Pretoria, South Africa
[3] Department of Physiology, Faculty of Health Sciences, School of Medicine, University of Pretoria, South Africa
[4] Proteomics Unit, Central Analytical Facility, Stellenbosch University, Stellenbosch, South Africa
[5] Bioengineering and Integrated Genomics Group, Next Generation Health Cluster, Council for Scientific and Industrial Research, Pretoria, South Africa

* Correspondence: THurrell@csir.co.za ; Tel.: +2712 841 4507

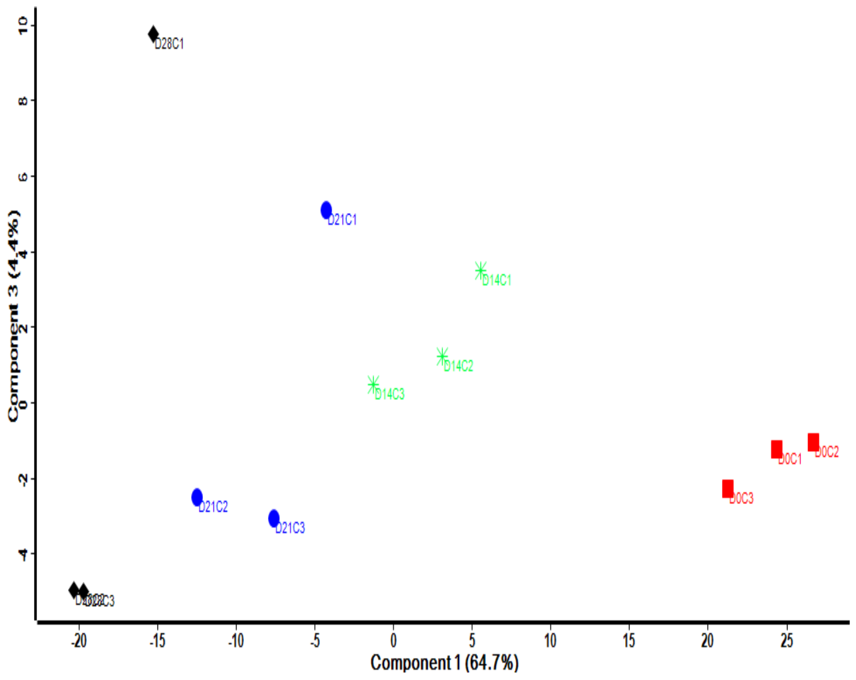

Supplementary Fig 1: PCA of HepG2 cell monolayers (red) and spheroids at Days 14 (green), 21 (blue), and 28 (black) comparing Component 1 versus Component 3. Figure generated using Perseus v. 1.6.7.0 software [28].

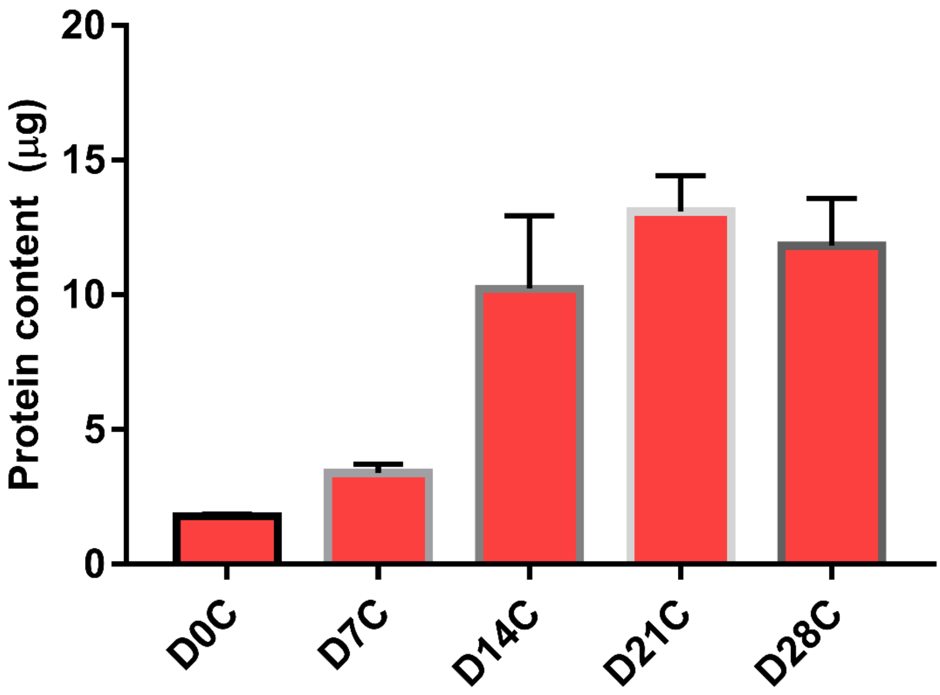

Supplementary Fig 2: Protein content per spheroid (mean ± SEM), used as an inference for cellular biomass, measured over the 28-day time course. Generated using GraphPad Prism windows software V7.0.0 (www.graphpad.com).

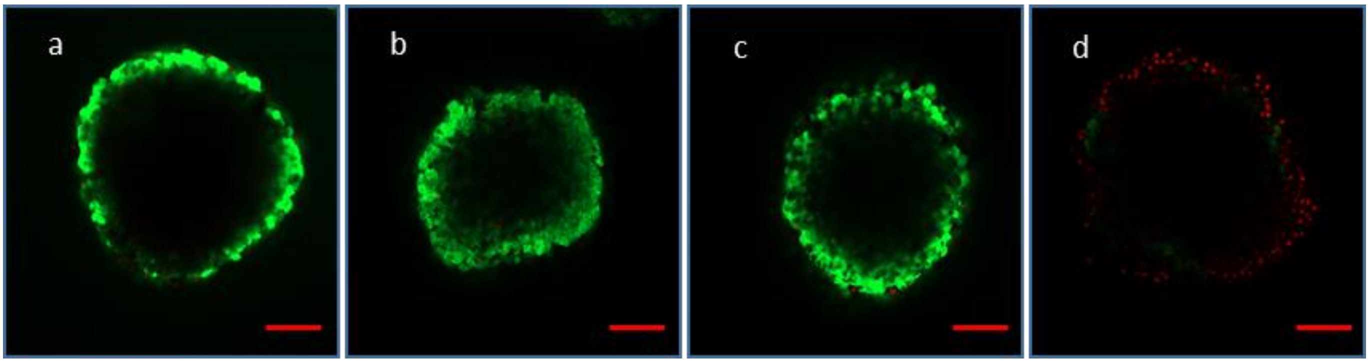

Supplementary Fig 3: Confocal micrographs of spheroids stained with FDA (green) and PI (red) at Days 14 (a), 21 (b), 28 (c), and Day 28 positive control exposed to 10 µg/mL Puromycin (d), Scale bars 100 µm. Generated using Zeiss Zen Blue software 3.0 (https://www.zeiss.com/microscopy/int/products/microscope-software/zen.html).

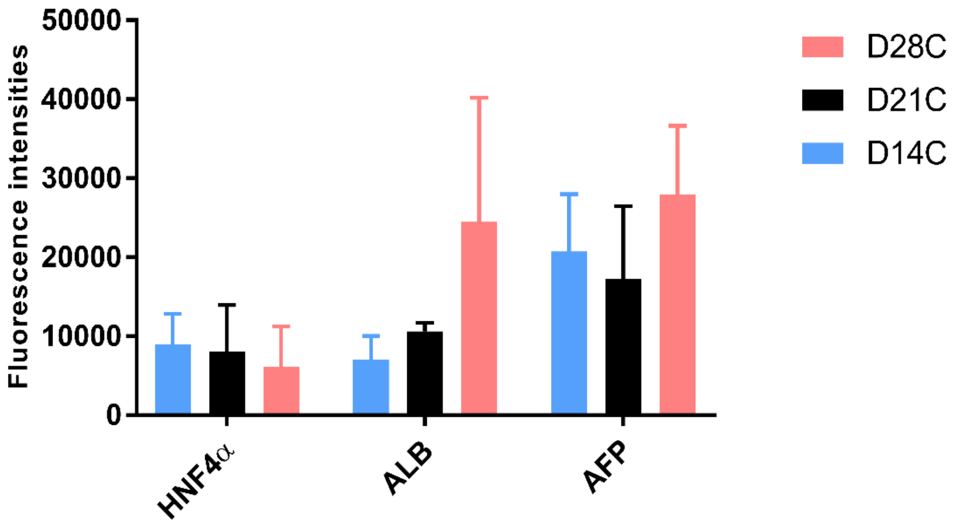

Supplementary Fig 4: Quantified relative fluorescent intensities (mean ± SD) of spheroids labelled with hepatic nuclear factor 4 alpha (HNF4α), albumin (ALB) and alpha-fetoprotein (AFP) normalised to DAPI. Generated using GraphPad Prism Generated using GraphPad Prism windows software V7.0.0 (www.graphpad.com).

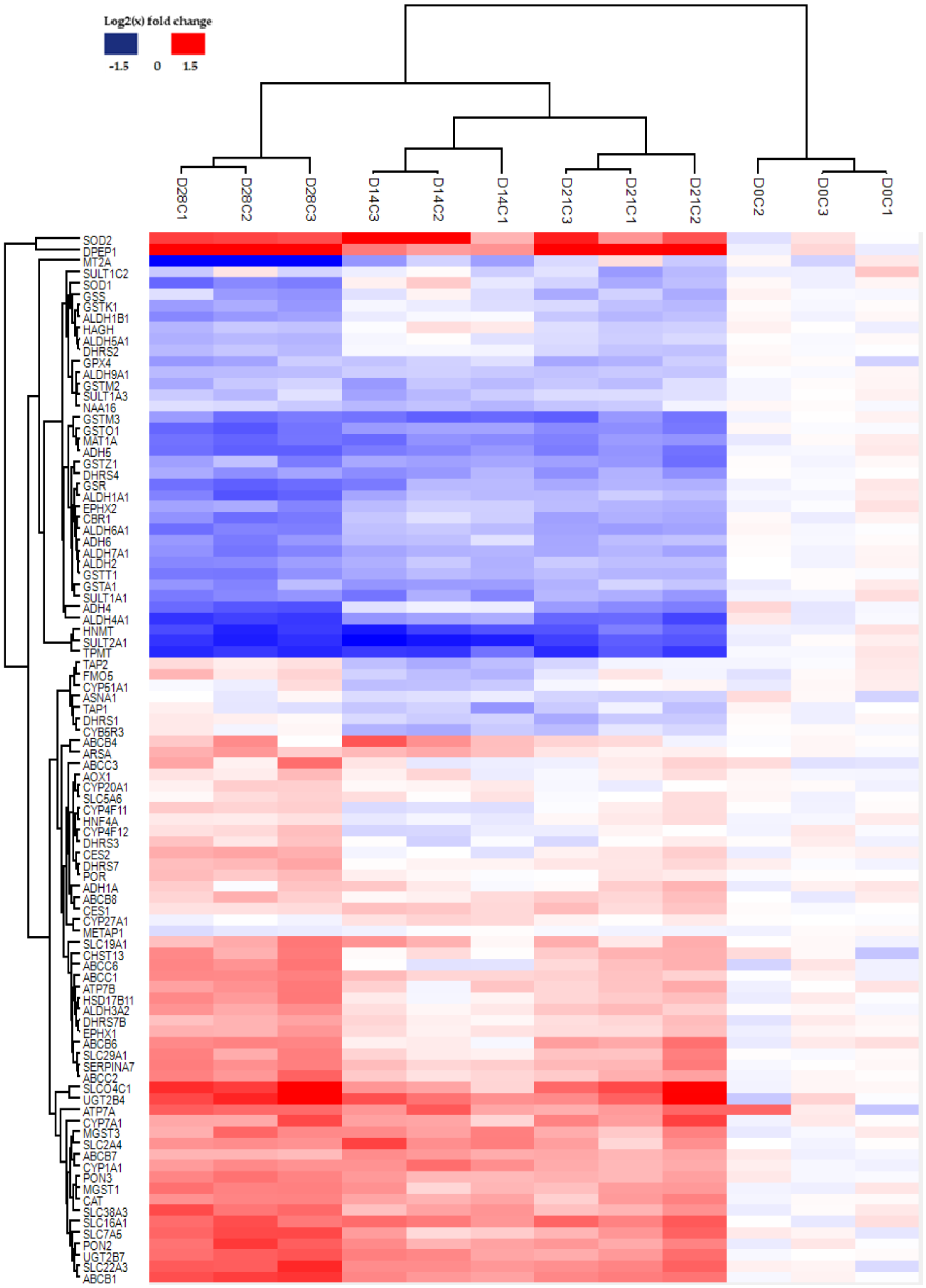

Supplementary Fig 5: Hierarchical clustering of proteins involved in ADME processes. Generated using Perseus v. 1.6.7.0 software.

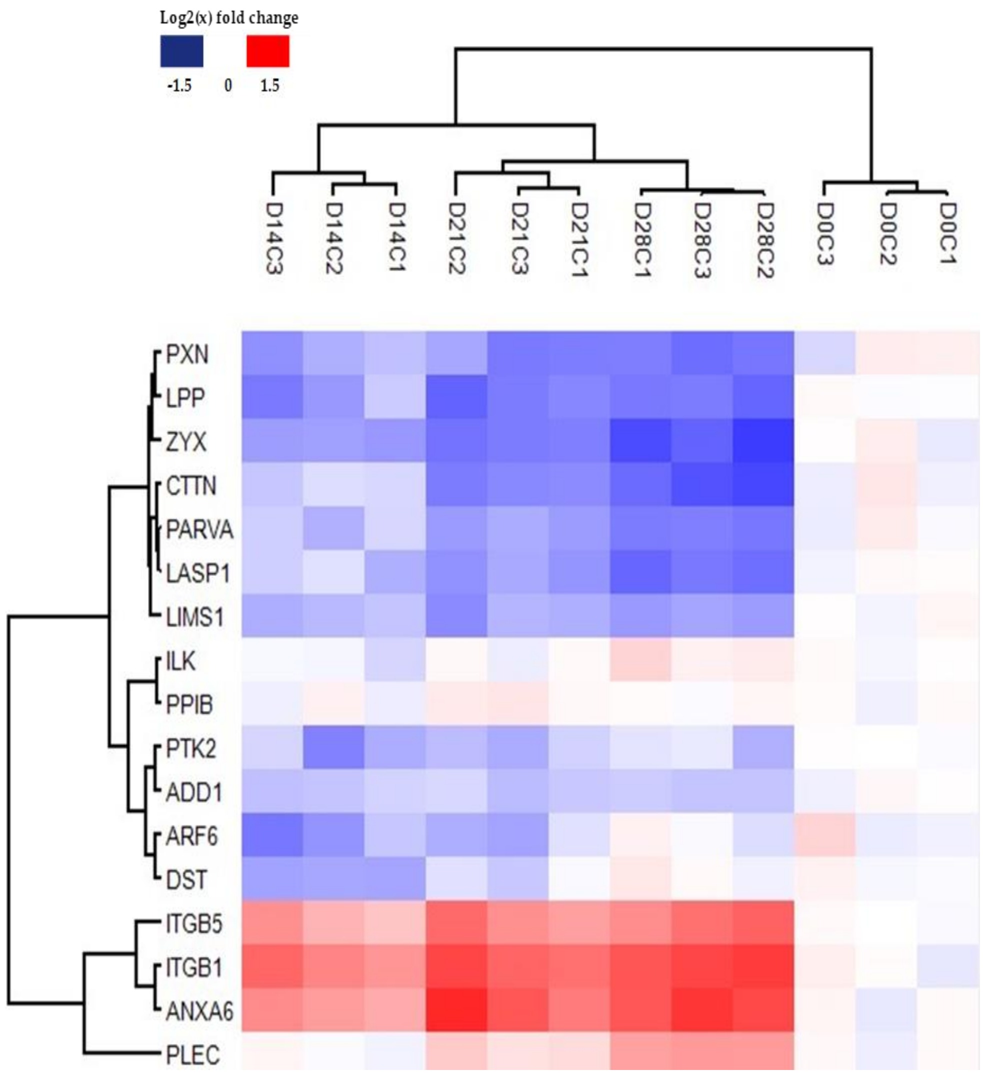

Supplementary Fig 6: Hierarchical clustering of proteins involved in Focal Adhesions (GO:0005925). Generated using Perseus v. 1.6.7.0 software.

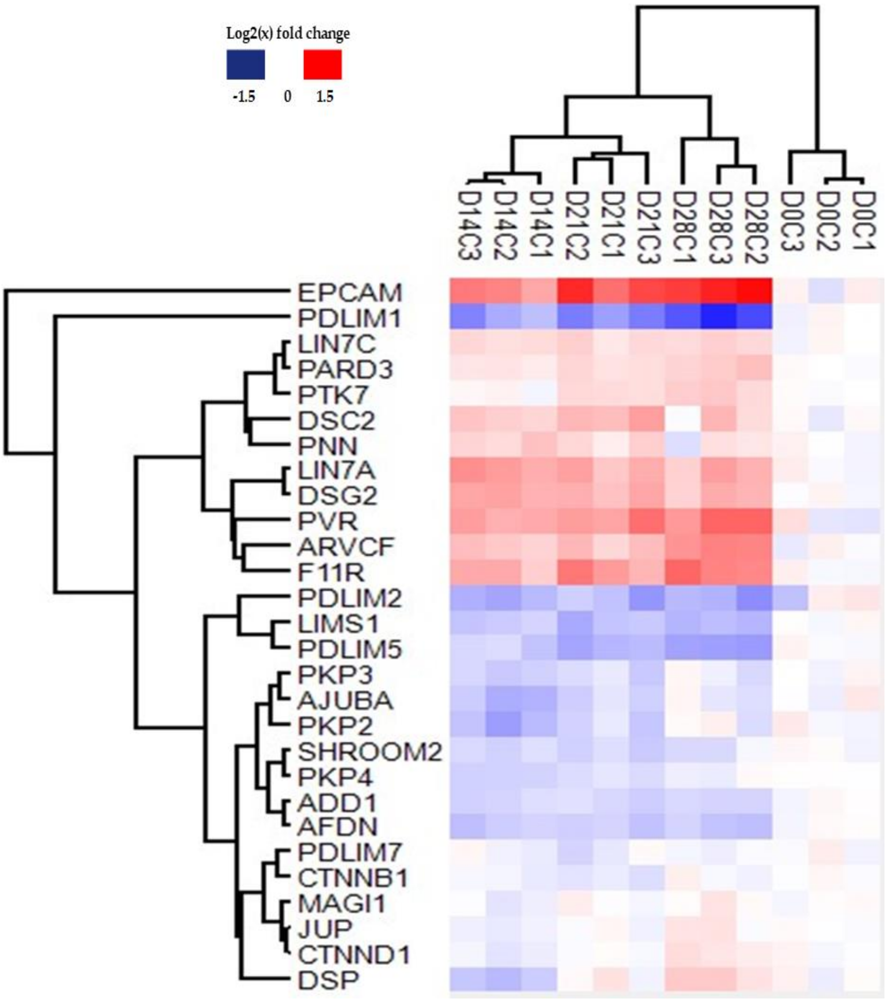

Supplementary Fig 7: Hierarchical clustering of proteins involved in cell-cell junctions (GO 0005911). Generated using Perseus v. 1.6.7.0 software.

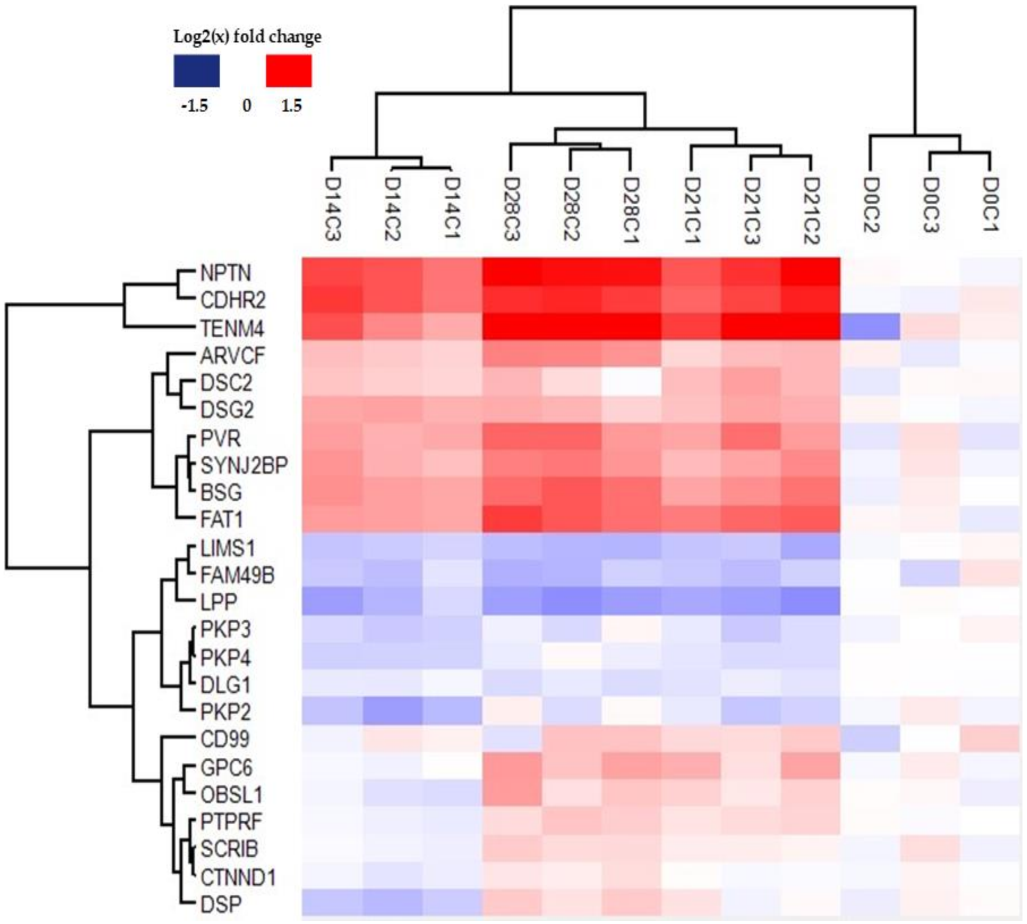

Supplementary Fig 8: Hierarchical clustering of proteins involved in cell-cell adhesion (GO 0098609). Generated using Perseus v. 1.6.7.0 software.
